# Sexual and reproductive health knowledge of secondary school adolescents in Fako, Cameroon

**DOI:** 10.11604/pamj.2022.41.340.31686

**Published:** 2022-04-27

**Authors:** Rita Muso Fubam, Nicholas Tendongfor, Oladapo Olayemi, Akin-Tunde Ademola Odukogbe

**Affiliations:** 1 Pan African University Life and Earth Sciences Institute, University of Ibadan, Ibadan, Nigeria,; 2 Department of Obstetrics and Gynaecology, College of Medicine, University of Ibadan, University College Hospital, Ibadan, Nigeria,; 3 Department of Public Health and Hygiene, University of Buea, Buea, Cameroon

**Keywords:** Adolescent, Cameroon, schools, sexuality, students, reproductive health

## Abstract

**Introduction:**

the correct sexual and reproductive health knowledge of adolescents remains important to empower them for healthy decision-making. The study aimed to assess the sexual and reproductive health knowledge of secondary school adolescents in Fako, Cameroon.

**Methods:**

a cross-sectional survey of 1180 adolescents from nine schools in Fako, was conducted using a structured interviewer - guided questionnaire. Data were analysed using SPSS version 26. Descriptive statistics and logistic regression analysis were used to outline knowledge and to identify predictors of knowledge respectively. Statistical significance was set at p<0.05.

**Results:**

more than half (54.0%) of the participants had overall good sexual and reproductive health knowledge. However, 63.1% and 55.3% of the participants had poor knowledge on reproductive system functions and sexually transmissible infections respectively. In addition, 56.0% had overall good contraceptive knowledge, with 51.6% having poor knowledge on condom. Being male (AOR=0.43, 95% CI=0.20, 0.92) and using the internet to search for sexuality related information (AOR=0.46, 95% CI= 0.22, 0.94) were associated with good knowledge. Being in lower secondary school was an independent predictor of poor knowledge (AOR= 3.83, 95% CI= 1.67, 8.81).

**Conclusion:**

although slightly above half of adolescent secondary school students had good sexual and reproductive health knowledge, there existed several gaps in such knowledge. Policymakers especially in the education sector need to evaluate the current state of school-based sexual and reproductive health education in Cameroon, in order to design comprehensive curricula, that will begin from lower secondary school. Internet-based comprehensive sexuality education is also needed.

## Introduction

The health of adolescents especially their sexual and reproductive health (SRH) remains crucial for the attainment of any sustainable development. Many behaviours acquired as adolescents can last a lifetime and affect their well-being and consequently that of the nation´s future adults. Risky behaviours that begin in adolescence account for a great proportion of preventable deaths that occur in adults [1]. A healthy transition of young people into adulthood can enable them use well the opportunities they have as adults. Therefore, investing in the sexual and reproductive health knowledge and health of adolescents is harnessing into a huge demographic dividend, especially in sub-Saharan Africa (SSA) where this age group constitutes a great proportion of the population [2,3].

Although there is an increase in the number of reproductive health risks and their negative outcomes among adolescents and the burden of communicable diseases in the developing countries, reproductive health knowledge and the adoption of safe sex behaviours remain low [4]. Being an extremely vulnerable group of people, adolescents especially in SSA continue to face barriers to sexual and reproductive health services. This has implications for the knowledge and skills needed by these young people to make decisions regarding their health. Evidence of the inattention to their sexual and reproductive needs is the disproportionately high rates of public health-related problems such as adolescent pregnancy, unsafe abortion and sexually transmissible infections including HIV/AIDS, sexual abuse and violence, and coercion among young people globally. It is estimated that 21 million girls aged 15 to 19 years and 2 million girls aged under 15 years become pregnant in developing regions and 23 million girls aged 15 to 19 years in this regions have an unmet need for modern contraception [5]. In Cameroon, adolescent pregnancy is still a major public health challenge with a national prevalence of 14.2% [6]. According to the U.S. President´s Emergency Plan for AIDS Relief (PEPFAR), the HIV prevalence in Cameroon among people 15-49 years in 2018 was 3.4% with about 2% of young people 15-24 years living with HIV [7]. These have implications for not only their physical wellbeing but social and mental well-being as well and the consequences go beyond the adolescents to the society as a whole. Although not self-sufficient, knowledge remains essential in guiding human behaviour and decision-making [8]. Therefore, sexual and reproductive health knowledge is vital for healthy decision making regarding sexual and reproductive health among adolescents. In addition, the environment in which young people grow today continues to change rapidly with fast growth in the technology and information sectors. With increased activity on social media platforms such as Instagram, Facebook, and Twitter, adolescents are more than ever, exposed to information and behaviours that can trigger risky behaviours [9]. While it may not be possible to fully censor information and sites young people are exposed to from different sources, it is important to provide them with adequate knowledge which can help them correctly evaluate any information to which they are exposed.

Although many studies have been conducted to assess the knowledge of adolescents on the Human Immunodeficiency Virus/Acquired Immune Deficiency Syndrome (HIV/AIDS) [10-13], other domains such as reproductive system and functions, other sexually transmissible infections (STIs) apart from HIV have not been considered in-depth. HIV/AIDS, although a long-standing global public health problem, the attention paid to it disproportionately affects the attention paid to other potentially dangerous STIs like HPV, Chlamydia and Hepatitis B infections. In Fako, Cameroon and other places, studies abound on knowledge of HIV/AIDS, mostly on senior secondary school adolescents [11-13] and university female students [14]. Those including students in lower secondary schools and assess more comprehensive reproductive health knowledge apart from HIV/AIDS are sparse. The risks associated with the unhealthy sexual behaviour of students in lower secondary schools who usually are of younger ages are important. These younger adolescents being uninformed unlike those in upper secondary schools are often victims of different forms of sexual assault and abuse. Comprehensive sexuality education has been widely recommended in improving adolescent sexual and reproductive health knowledge and behaviour [15,16]. However, in designing such programs or improving existing ones, identifying gaps in knowledge is a priority. It is necessary, therefore, to determine the comprehensive knowledge that adolescents including younger adolescents have in order to ensure strategies that are targeted.

The aim of this study was to assess the sexual and reproductive health knowledge of secondary school adolescents in Fako, Cameroon and to find out if there were any gaps in their knowledge. It also sought to identify the determinants of sexual and reproductive health knowledge among these adolescent students.

## Methods

**Study design:** this was a descriptive cross-sectional study.

**Study area and setting:** this study was conducted in Fako division, in the South West Region of Cameroon from November 2020 to January 2021. Fako is the most populous and urban of the six divisions of the region and is considered an education hub in the region, with numerous schools at all levels and attracting many young people from across the country. It is composed of four sub-divisions including Buea, Tiko, Limbe and Muyuka. The Muyuka sub-division, which has been most significantly affected by ongoing socio-political crises with grave socioeconomic disruptions, was excluded from this study. There are about 65 secondary schools in Fako, consisting of public, faith-based and lay private schools. The majority of the schools are day schools and coeducational. In addition, schools in Fako offer different kinds of education mainly general, technical and commercial education. Secondary schools in Fako are to a great extent similar in structures, facilities and services to other secondary schools across the national territory.

**Study population:** this study included secondary school adolescents aged 12-19 years in Fako, Cameroon. Participants were chosen from nine randomly selected co-educational secondary schools in Fako. In addition, secondary schools included were those that had both lower (Form One to Form Five [6^th^ to 10^th^ grades]) and upper secondary school (Lower and Upper Sixth [11^th^ and 12^th^ grades]).

**Sample size calculation:** the sample size was obtained using the formula for estimating proportion in a cross-sectional study:


$$n=\frac{Z^{2}p(1-p)}{d^{2}}$$

Where n is the sample size, Z is the value of standard normal deviation to the level of confidence, p is the expected prevalence expressed in proportion and d is the absolute precision. In a study carried out to assess the knowledge, attitudes and practices regarding HIV/AIDS among senior secondary school students in Fako, Cameroon, 37.1% had inadequate knowledge on how HIV/AIDS (12). This was used as a proxy for sexual and reproductive health knowledge among in-school adolescents in Fako, with an absolute precision of 3% (0.03) and Z=1.96 for a confidence level of 95%. The minimum sample calculated was 1006. Assuming a possible non-response rate of 10%, the sample final sample size for the study was 1180.

**Sampling technique:** a multistage cluster random sampling technique was used to select the sample of students (males and females) from nine randomly selected schools which included five public schools, two faith-based schools and two lay-private schools. In the first stage, all the streams of each class in each of the selected schools were listed as clusters in each school; this was followed by a random selection of clusters that were included in the study. The sample size was proportionately distributed to the selected schools after a total number of students in each school was obtained from the administration. The number of students to be selected from each cluster was calculated based on the study sample of the school and the number of clusters in that school. A random selection of numbers from among these students was done to select the calculated number of participants for each cluster.

**Study variables:** the main outcome variable was sexual and reproductive health knowledge, considered in four main domains addressing specific areas. These included reproductive function knowledge (5 items), knowledge on HIV and other STIs (8 items), contraceptive knowledge (6 items), and condom knowledge (5 items). Questions assessing knowledge were provided “Yes” and “No” or “True” and “False” responses. “Not sure” responses were included in some instances to minimize any guessing. Knowledge scores were assessed for the domains by assigning “1” for each correct response. Incorrect responses or “Not sure” responses were assigned “0”. A composite score was calculated for each domain and the mean score was used as a cut-off. Participants who had below the mean score for any domain were categorized as having poor knowledge, while those above the mean score were considered to have good knowledge. To obtain the overall knowledge score, a composite score was obtained by summing up the scores of all four domains. The final scores were converted to percentages and considering a cut-off of 50%, the overall knowledge score was dichotomised to poor knowledge (less than or equal to 50%) and good knowledge (above 50%). Explanatory variables included sociodemographic, socioeconomic characteristics and sexuality-related variables.

**Data collection tool and process:** the data collection tool was adapted from the World Health Organization´s Illustrative Questionnaire for Interview-Surveys with Young People by John Cleland. This instrument has been used in several countries including low and middle-income countries like Nigeria, Kenya, Tanzania, Nicaragua and India, to assess sexual and reproductive health needs in young people [17]. The instrument was modified with respect to the objectives of the current study and study setting. A pilot study was conducted in a similar group of students prior to the main study and ambiguous questions were clarified in the final copy of the instrument. The school administrations of the selected schools were contacted for permission, once this was obtained, the principal investigator worked closely with a teacher(s) assigned to assist the research team. They worked to identify convenient times and appropriate spaces to conduct the interviews without disrupting learning activities. In each school, eligible students were administered the interviewer-guided instrument. This was done by eight trained research assistants in the English language and to ensure completeness, the research team cross-checked all questionnaires before leaving each school.

**Data analysis:** data were entered using Epi Info version 7 and statistical analysis was performed with SPSS version 26.0. Descriptive analysis was performed on sociodemographic and sexuality-related variables for frequencies and percentages and central tendencies (means and standard deviations). Descriptive analysis was also performed on the sources of sexual and reproductive health information and on the different knowledge domains to identify areas of gaps. Bivariate analysis was performed using simple logistic regression to identify potential predictors of sexual and reproductive health knowledge. Variables with p-values < 0.2 [18] were fitted into a multiple logistic regression model to identify predictors independently associated with poor knowledge. Statistical significance was set at p<0.05.

**Data availability statement:** the data that support the findings of this study are available from the corresponding author upon reasonable request.

**Ethical considerations:** this study was approved by the University of Ibadan/University College Hospital, Ibadan (UI/UCH) Ethics Committee (UI/UCH-EC: 017/20). Administrative approvals were obtained from Cameroon´s South West Regional Delegations of Secondary Education (SD2020 / 061 / MINSEC / RDSW / SDGA / SPS) and Public Health (R11 / MINSANTE / SWR / RDPH / PS / 615 / 780). Permission was obtained from the Principals of the selected schools. Informed consent was obtained from the guardians and assent from participants before questionnaires were administered. This was after participants were briefed on the objectives of the study and assured on confidentiality and anonymity of their responses. Interviews were conducted under conditions that optimised confidentiality and the privacy of participants.

## Results

**Sociodemographic characteristics of study participants:** a total of 1180 adolescent students took part in the study. The average age of participants was 15.43 + 2.13 years, with majority (63.8%) of them aged between 15 to 19 years. More than half (56.0%) of the participants were females and 65% of participants were in the lower secondary school. About 62% were enrolled in public schools, with about 68% in general education. A majority (92.8%) of the participants were of the Christian religion and none of them was married. More than half (55.2%) of the participants lived with both parents while 59.6% lived in households with more than five persons (Table 1).

**Table 1:** sociodemographic characteristics of study participants

Variables	Frequency (n)	Percentage (%)
**Age group (in years)**		
12-14	427	36.2
15-19	753	63.8
**Mean (±SD)**	15.48 (2.13)	
**Gender**		
Male	519	44.0
Female	661	56.0
**Class category**		
Lower secondary school	767	65.0
Upper secondary school	413	35.0
**Type of school**		
Public school	727	61.6
Private school	233	19.7
Faith-based school	220	18.6
**Kind of education**		
General Education	800	67.8
Technical Education	184	15.6
Commercial Education	196	16.6
**Religion**		
Christian	1095	92.8
Muslim and others	85	7.2
**Marital status**		
Single (never married)	1180	100.0
**Household composition**		
Living with single parent	345	29.2
Living with both parents	651	55.2
Others	184	15.6
**Number of people in household**		
Five or less	477	40.4
More than five	703	59.6

**Sexuality-related characteristics of study participants:** as shown in Table 2, less than half (40.4%) of the adolescents had sexuality-related discussions with family members. Although three fifths (63.1%) had access to the internet, less than half (45.4%) used the internet to search for sexuality-related information. Sixty-three per cent of the participants had ever attended a class in school on sexual and reproductive health-related topics and three quarters (74.7%) of them indicated the need for more such classes in schools. A fifth (19.9%) of the participants had ever had sex, with an average age at sexual debut of 14.93 + 2.13 years (ranging between 5 to 19 years).

**Table 2:** sexuality-related characteristics of participants

Variables	Frequency (n)	Percentage (%)
**Discussion with family members on sexuality-related information**		
Yes	478	40.5
No	702	59.5
**Access to internet**		
Yes	744	63.1
No	436	36.9
**Use internet for sexuality-related information**		
Yes	338	45.4
No	406	54.6
**Attends clubs/parties**		
Yes	463	39.2
No	717	60.8
**Attended clubs within past one month**		
Yes	297	64.1
No	166	35.9
**Consumes alcohol**		
Yes	326	27.6
No	854	72.4
**Consumed alcohol within past one month**		
Yes	245	75.2
No	81	24.8
**Attended class in school on SRH**		
Yes	743	63.0
No	437	37.1
**Preferred frequency of SRH classes**		
More	555	74.7
Less	42	5.7
Just okay	146	19.7
**Ever had sex**		
Yes	235	19.9
No	945	80.1
**Age at first sexual intercourse**		
Mean(29)	14.93(2.13)	
Minimum	5	
Maximum	19	

**Figure 1:**
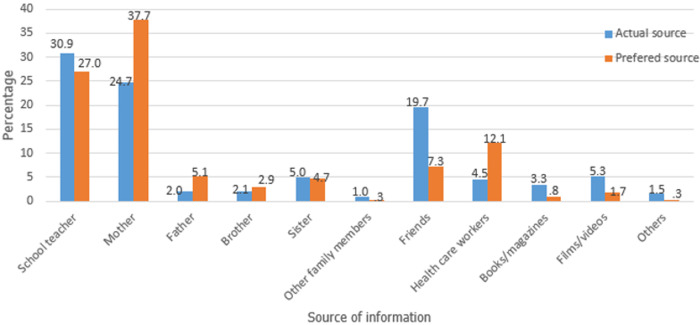
sources of sexuality-related information

**Sources of reproductive health information:** as represented in Figure 1, schoolteachers (30.9%) were the main source of information concerning puberty, the reproductive system and relationships between boys and girls. This was followed by mothers (24.7%) and friends (19.7%). However, most of the adolescents would prefer mothers (37.7%) as a primary source of such information, followed by teachers (27.0%) and healthcare workers (12.1%).

**Sexual and reproductive health knowledge:** Table 3 shows that after computing the scores for the different domains, 63.1% of the participants had poor knowledge of reproductive system functions. All the participants were aware of HIV/AIDS and 54.8% had a good knowledge on HIV/AIDS. When considered with other STIs, more than half 55.3% of the participants had poor knowledge on STIs as a whole. Over four-fifths, (88.6%) of the participants were aware of contraceptives, with more than half (56.0%) of them having good contraceptive knowledge. This knowledge was assessed by summing up the scores of their awareness of some contraceptive methods including pills (52.9%), injections (50.1%), condoms (94.6%), emergency contraceptive pills (56.7%), withdrawal method (50.7%) and periodic abstinence (64.8%). A small proportion (2.7%) of those who were aware of contraceptives could identify other modern methods such as tubal ligation, intrauterine devices and implants. Worthy of note is another small proportion (6.2%) who identified wrong methods of contraception such as taking whisky, salty water, lemon and honey after sexual intercourse. Over four fifths (84.7%) of the participants were aware of condom, with more than half (51.6%) of them having a poor knowledge on condom; 50.2% of the respondents had a negative attitude regarding condoms.

**Table 3:** sexual and reproductive health knowledge

Variables	Frequency	Percentage (%)
**Reproductive function knowledge**		
Poor knowledge	744	63.1
Good knowledge	436	36.9
**HIV/AIDS awareness**		
Yes	1180	100.0
**Know other STI**		
Yes	854	72.4
No	326	27.6
**HIV/AIDS Knowledge**		
Poor Knowledge	533	45.2
Good knowledge	647	54.8
**HIV/AIDS and other STIs knowledge**		
Poor knowledge	653	55.3
Good knowledge	527	44.7
**Contraceptive awareness**		
Yes	1046	88.6
No	134	11.4
**Contraceptive knowledge(n=1046)**		
Poor contraceptive knowledge	460	44.0
Good contraceptive knowledge	585	56.0
**Condom awareness**		
Yes	1000	84.7
No	180	15.3
**Condom knowledge(n=1000)**		
Poor knowledge	516	51.6
Good knowledge	484	48.4
**Condom attitude (n=1000)**		
Negative attitude	502	50.2
Positive attitude	498	49.8
**Composite SRH knowledge score**		
Poor SRH knowledge	543	46.0
Good SRH knowledge	637	54.0

**Determinants of sexual and reproductive health knowledge:** the bivariate analysis presented in Table 4 shows that there was significantly poor knowledge with decreasing age (COR=4.67, p=0.00) and among those in lower secondary school (COR=4.41, p=0.00). Being male (COR=0.67, p=0.00) and attending public (COR= 0.67, p=0.01) and private schools (COR= 0.40, p=0.00) schools had a significant positive effect on knowledge. Having sex-related discussions with family members (COR=0.57, p=0.00), using the internet to look for sexuality-related information (COR=0.42, p=0.00), attending a SRH class in school (COR= 0.50, p= 0.00) also had significant positive effects on knowledge. Those who had ever had sex before (COR=0.28, P=0.00) were also less likely to have poor knowledge. In the adjusted model, there was a significant decrease in the odds of having poor knowledge with being male (AOR=0.43, 95% CI= 0.20, 0.92). Being in lower secondary school was also a determinant of poor knowledge, with the odds of having a poor knowledge being about four times higher in those in lower secondary school (AOR= 3.83, 95% CI= 1.67, 8.81). There was a significant positive effect of using the internet to look for sexuality-related information on SRH knowledge, with a decrease odd of having poor SRH knowledge among students who used the internet to look for sexuality-related information (AOR=0.46, 95% CI= 0.22, 0.94).

**Table 4:** factors associated with sexual and reproductive health knowledge

Variable	Level	OR (95% CI)	p-value	AOR (95% CI)	p-value
**Age (years)**	12-14	4.67(3.60-6.06)	0.00	1.72(0.63-4.68)	0.29
	15-19	1			
**Sex**	Male	0.67(0.53-0.85)	0.00	0.43(0.20-0.92)	0.03*
	Female	1		1	
**Class**	Lower secondary school	4.41(3.40-5.73)	0.00	3.83(1.67-8.81)	0.00*
	Upper secondary school	1		1	
**School type**	Public school	0.65(0.48-0.89)	0.01	0.79(0.24-2.61)	0.69
	Private school	0.40(0.27-0.58)	0.00	0.55(0.16-1.84)	0.33
	Faith-based school	1		1	
**Kind of education**	General education	0.76(0.55-1.03)	0.08	0.79(0.27-2.35)	0.68
	Technical education	0.89(0.59-1.33)	0.57	0.1.27(0.30-5.40)	0.75
	Commercial education	1		1	
**Religion**	Christian	0.73(0.47-1.14)	0.17	0.54(0.15-1.89)	0.33
	Muslim/others	1		1	
**Household arrangement**	Single parent	1.22(0.86-1.75)	0.27	1.43(0.50-4.09)	0.51
	Both parents	1.32(0,95-1.84)	0.10	0.88(0.31-2.48)	0.81
	Others	1			
**Number of persons/household**	Five or less	1.02(0.81-1.29)	0.86	-	-
	More than five	1			
**Sex-related discussions with family**	Yes	0.57(0.45-0.72)	0.00	0.98(0.48-1.99)	0.95
	No	1		1	
**Obtains sex-related information from internet**	Yes	0.42(0.31-0.57)	0.00	0.46(0.22-0.94)	0.03*
	No	1		1	
**Attended club in the last month**	Yes	0.69(0.47-1.01)	0.05	1.13(0.44-2.86)	0.80
	No	1		1	
**Consumed alcohol in the last month**	Yes	0.68(0.41-1.14)	0.15	0.58(0.23-1.50)	0.26
	No	1		1	
**Attended SRH class in school**	Yes	0.50(0.39-0.63)	0.00	1.37(0.63-3.01)	0.43
	No	1		1	
**Ever had sex**	Yes	0.28(0.20-0.38)	0.00	0.50(0.24-1.06)	0.07
	No	1		1	

OR-Crude Odd Ratio AOR-Adjusted Odd Ratio CI-Confidence Interval “1”- Reference Category

## Discussion

This study was conducted to assess the sexual and reproductive health knowledge of adolescent students in Fako, Cameroon. It revealed that slightly above half of adolescent secondary school students in Fako had an overall good sexual and reproductive health knowledge. Similar findings were reported in a study in Nigeria in which more than half of the adolescents were knowledgeable on sexual and reproductive health [19]. Contrary findings have however been reported in another study in which adolescents had suboptimal SRH knowledge [20]. There were gaps in the knowledge on reproductive functions, STIs and condom use. Similar findings were reported in a review of sexual and reproductive health knowledge among eight sites in sub-Saharan Africa [21]. Wirsiy *et al*. [22] reported a higher proportion (78.6 %) of adolescent girls who had incorrect knowledge on HIV and other STIs. The gaps reported in this study may be partly explained by the wide variety of adolescent students included in the study, from technical and commercial education schools who usually are not taught subjects such as biology, in which some aspects of reproduction are taught. The contraceptive knowledge of students in this study was good (56.0%) contrary to findings of another study in which there was poor knowledge of contraceptive methods [23]. In this study, contraceptive knowledge was assessed by correct identification of some traditional and modern contraceptive methods, with a great majority being aware of a method like condom and over half knowing other methods.

Female adolescent students had significantly poor sexual and reproductive health knowledge compared to the male adolescents. This finding is consistent with studies among adolescents in Nigeria [19], Nicaragua [23], India [24], Zambia [20]. Although there is an increase in the attention paid to gender imbalances in Cameroon, there continue to be certain restrictions on the female. This may affect how they report their knowledge regarding sexuality in order not to be considered promiscuous. This information is particularly important for decision makers who must consider other educational programs that target females apart from general sexuality education which includes both sexes. Females have higher risks from sexually transmitted diseases and other sexuality-related problems including unwanted pregnancies.

Students in lower classes also had statistically significant poor knowledge compared to those in the senior classes. This is consistent with findings from India in which higher standard students were more knowledgeable than lower ones [24]. Subjects like biology and human biology taught in schools, that have aspects of sexuality are usually not considered with details in the lower classes. In addition, many school programs that provide sensitization on sexuality-related topics in schools have often focused on higher classes. The reason for this is the consideration that those in upper classes are more mature with more need for such information. Although age was not a predictor of knowledge, older adolescents who are mostly in upper secondary school may have had more exposures and experiences that may have influenced their knowledge.

In the present study, the use of internet to search for sexuality-related information was found to be a significant positive predictor of sexual and reproductive health knowledge. This is comparable to other studies in which knowledge increased significantly among those who received internet- based sex education [19,25]. The internet undoubtedly has a wealth of information for its users, making it a teacher and a readily available one, therefore a source of education not only for adults but also for younger people. On the other hand, some information provided on the internet may neither be age-appropriate nor evidenced-based [26]. There is therefore need for well-designed evidence-based programs on the internet, education of young people on healthy internet use, and its regulation and supervision.

The study revealed that schoolteachers were common sources of sexuality-related information to adolescent students. Similar findings have been reported in other studies [22-24]; the content of such information in the current study was however, not assessed, an area which needs to be considered in future studies. The students preferred to obtain information related to sexual and reproductive health from their mothers as also reported in previous studies [27]. Young people often consider their parents trustworthy and believe that parents have their best interest at heart. There is need for enhancing parent-adolescent sexuality communication [28]. This will help prevent adolescents from turning to sources such as friends or the internet where they may get incorrect information. This can also provide them with opportunities to verify information they obtain from other sources.

**Strengths and limitations:** an important strength of this study is the large sample size with the inclusion of all types of secondary schools in Cameroon. Furthermore, including both students of the lower and upper secondary school levels widened the age range of adolescents included in this study. This study was not void of limitations; considering that responses were self-reported, social desirability bias cannot be ruled-out, common with research involving socially judged behaviours. This may have affected the responses provided by these adolescent students. However, attempts to minimize this was by ensuring that the questionnaires were anonymous and completed in conditions that maximized privacy. This study included only secondary school-going adolescents and schools in the urban areas of Fako, hence cannot be generalised to all adolescents in Fako. Notwithstanding, policymakers especially in the education sector and sexual and reproductive health program designers will find the results of this study useful.

**Implications of findings:** the main aim of this study was to assess the sexual and reproductive health knowledge of secondary school adolescents in Fako, Cameroon. The findings of this study provide empirical data on the level of knowledge adolescent students possess concerning their sexual and reproductive health and the factors associated with this knowledge. This information is important, as it will allow policy-makers and program designers and other stakeholders involved with the health of adolescents to design informed programs and take actions accordingly. Another important implication of this study derives from identification of areas of gap in the knowledge these adolescents have which can help program and curriculum designers to know which areas that need closer attention as they educate these adolescents.

## Conclusion

The findings of this study indicated that slightly above half of adolescent secondary school students had good sexual and reproductive health knowledge. However, there existed several gaps in such knowledge. Also being female and in lower secondary were associated with poor knowledge while the use of the internet to search for sexuality-related information was associated with good sexual and reproductive health knowledge. The government through the Ministry of Secondary Education should evaluate the state of comprehensive sexual and reproductive health education in schools, design and implement age-appropriate formal programs including curricula, which beginning from the lower levels of education. In addition to a general sexual and reproductive health education program, programs that improve the knowledge of females are needed. Internet program designers should develop evidence-based educative programs that will improve comprehensive sexual and reproductive health education among adolescents.

### What is known about this topic

- Sexual and reproductive health knowledge remains essential in enhancing positive decision making among adolescents;
- Young people especially in developing countries continue to face barriers to sexual and reproductive health knowledge and services.

### What this study adds

- With increasing exposure and use of the internet by adolescents, internet-based sexuality education can be designed to provide age appropriate sexual and reproductive health knowledge to adolescents;
- School-based sexual and reproductive health programs should focus attention not only on senior secondary school students but students in the lower sections also.
